# Laser Scanning Measurements on Trees for Logging Harvesting Operations

**DOI:** 10.3390/s120709273

**Published:** 2012-07-05

**Authors:** Yili Zheng, Jinhao Liu, Dian Wang, Ruixi Yang

**Affiliations:** School of Technology, Beijing Forestry University, Beijing 100083, China; E-Mails: zhengyili@bjfu.edu.cn (Y.Z.); wangdian@bjfu.edu.cn (D.W.); yrx1006_c2@163.com (R.Y.)

**Keywords:** laser measurement, logging harvester, Fletcher-Reeves conjugate gradient, kinematics control

## Abstract

Logging harvesters represent a set of high-performance modern forestry machinery, which can finish a series of continuous operations such as felling, delimbing, peeling, bucking and so forth with human intervention. It is found by experiment that during the process of the alignment of the harvesting head to capture the trunk, the operator needs a lot of observation, judgment and repeated operations, which lead to the time and fuel losses. In order to improve the operation efficiency and reduce the operating costs, the point clouds for standing trees are collected with a low-cost 2D laser scanner. A cluster extracting algorithm and filtering algorithm are used to classify each trunk from the point cloud. On the assumption that every cross section of the target trunk is approximate a standard circle and combining the information of an Attitude and Heading Reference System, the radii and center locations of the trunks in the scanning range are calculated by the Fletcher-Reeves conjugate gradient algorithm. The method is validated through experiments in an aspen forest, and the optimized calculation time consumption is compared with the previous work of other researchers. Moreover, the implementation of the calculation result for automotive capturing trunks by the harvesting head during the logging operation is discussed in particular.

## 1. Introduction

Because the circumstances of forest areas are very complex and hazardous, it is very dangerous and laborious to harvest standing trees by hand-operated machines and tools. In the last decade, with the development of the hydraulic controls and sensor techniques, more and more logging harvesters are being used in forestry [1–3]. The logging harvester can complete successive tasks of felling, delimbing, measuring and bucking, and is very suitable for large scale clear cutting operations in fast growing plantations, as well as on hillsides. A comfortable work environment, easy maintenance, efficiency, less manpower, and many productive days of work can be expected when using the logging harvesters. This leads to lower costs and better price performance ratio of the logging operations.

The Forest and Environment Equipment Research Institute of Beijing Forestry University has been dedicated to the research on logging harvesters for years [4,5]. It was found by experiments on logging harvester prototypes that the processes of delimbing, peeling, and bucking can be completed fast, but for the process of the alignment of harvesting head to capture the trunk, dues to the blind areas of the operator and vibration of the cane and vehicles chassis, the operator has to perform repeated observations, judgments and operations, which lead to the time and fuel losses. It is therefore of significance to find an efficient method to measure the location of surrounding trees for the harvesting head to achieve automatic capture.

Laser scanner is a non-contact measurement system that can scan surroundings two or three dimensionally with a radial field of vision using infra-red laser beams. The distance between the laser scanner and the object is determined by the time of flight of laser light pulses: a pulsed laser beam is emitted and reflected if it meets an object. From those distance data, a point cloud is created describing the shapes of the objects surrounding the scanner. Laser scanning offers new possibilities in tree measurement applications in forestry.

Jutila, Kannas *et al.* [6] discuss a method for diameter and location measurement of tree parameters using a 2D laser scanner mounted on a mobile ATV platform. The error of the tree diameter calculations is 4%. Thies, Pfeifer *et al.* [7] used a 3D terrestrial laser scanner to capture the geometric aspects of a tree: the radius, length and diameter of the trunk and individual branches. Liang, Litkey *et al.* [8] presented a fully automatic stem-mapping algorithm using 3D single-scan terrestrial laser scanning data for collecting individual tree information from forest plots. Öhman, Miettinen *et al.* [9,10] used 2D scanning laser range finders, machine vision systems and GPS to get information about the surrounding forest, such as tree diameters, positions and stem density. This information can be used on-line for the simultaneous localization and mapping for the forest harvesters or off-line in a forest asset management system. Rossmann *et al.* [11] mounted two laser scanners on the right and left side of the logging harvester cabin, generating a local tree map from the point cloud data of the mounted laser scanners, and using a particle filtering matching algorithm to form the global tree map for wood harvesting.

In this paper, a low-cost 2D laser scanner and an inertial measurement system are mounted on the outer boom of the crane, and are used to obtain the point cloud of the surrounding trees. In Section 2, the whole laser measurement equipment is described, and a laser scanning experiment is carried out in an aspen forest; In Section 3, the hierarchical cluster algorithm and filtering algorithm are used to extract each trunk from the point cloud. The trunk radii and location of the trunks are calculated by the Fletcher-Reeves conjugate gradient algorithm; In Section 4, the measurement results are given and compared with previous work of other researchers; In Section 5, the implementation of the result for automated trunk capture is discussed. Our conclusions are presented in Section 6.

## 2. The Description of the Equipment

### 2.1. The Equipment Hardware

3D laser scanners are expensive and unsuitable for continuous measurements [6]. Accordingly, a LMS291 2D laser scanner from SICK Inc. is used as the primary sensor. The measurement data corresponding to the surrounding contour is scanned by the LMS291, and is output in binary format via the RS485 data interface at the rate of 10 Hz to form the raw point cloud. As a result of the beam geometry, the maximum space between two laser beams is related to the scanning angular resolution and maximum scanning range. To get a local tree map of adequate resolution for logging harvesting, the scanning angular resolution of the LMS2291 is set to 0.25°, the maximum scanning angle is 100°, and maximum scanning distance is 8 m.

**Figure 1.**
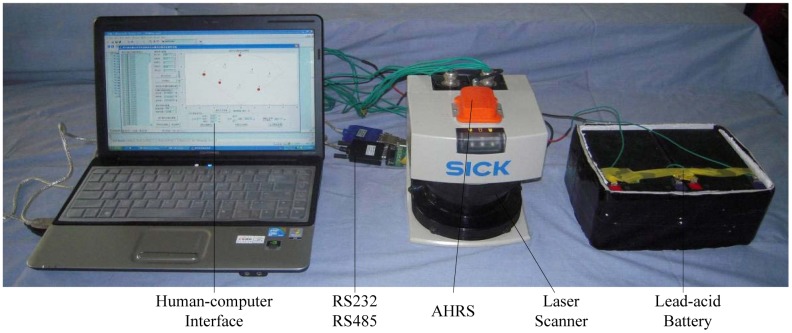
The whole hardware of measurement equipment.

The 2D laser scanner can be installed on the outer boom of the crane, but the tilt and orientation angle of the scanner plane should be known. A MTi Attitude and Heading Reference System (AHRS) of Xsens Inc. is used. AHRS is a miniature inertial measurement unit with integrated 3D magnetometers, and is fixed on the top of LMS291 as shown in Figure 1. AHRS is capable of outputting roll *α*, pitch *β* and yaw *γ* of the scanner plane in real time via the RS232 data interface. The electronics of LMS291 and AHRS are powered directly from a 24 V lead-acid battery. The data analysis software is implemented on the computer. The whole measurement equipment is shown in Figure 1.

### 2.2. The Data Analysis Flow of the Equipment

**Figure 2.**
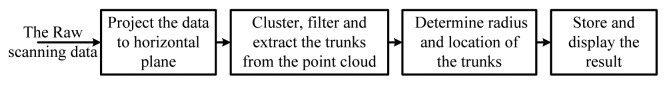
The data analysis flow.

The overall data analysis flow of the equipment for the measurement and calculation of the tree parameters is shown in Figure 2, and is mainly divided into four consecutive phases. The first phase is projecting the raw point cloud onto a horizontal plane according to the tilt angle *α* and *β* of the scanning plane. The second phase is filtering the invalid scanning data against some criteria, and extracting each trunk from the calibrated point cloud. The third phrase is determining the trunk radii and location of the trunks for the harvesting head. The fourth phrase is storing the results and displaying the useful information on the human-computer interface.

## 3. Extracting Trunks Feature for Logging Harvesting

### 3.1. Projecting the Raw Scanning Data

**Figure 3.**
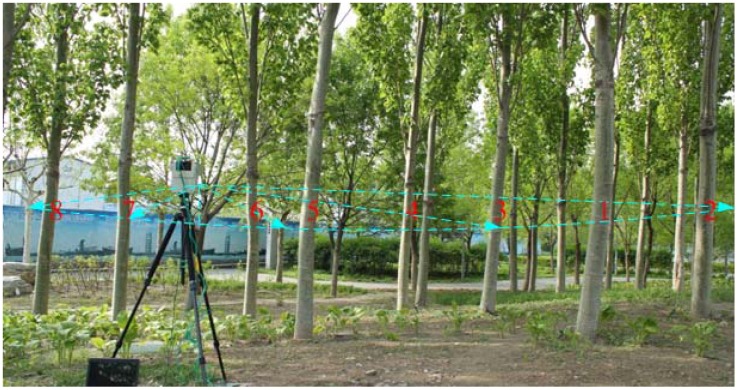
The experiment in the aspen forest.

In our experiments, the laser scanner was fixed on a tripod with telescopic legs as seen in Figure 3. Laser beams reflect if they meet the trunk or other object, and a fan-shaped scan is made of the surrounding area. Depending on the angular resolution of the LMS291, the distance value is provided every 0.25° from 40° to 140°, and the number of distance values is 401. As the individual distance values are given out in sequence via the RS485 data interface particular, the angular position of every individual distance value can be allocated on the basis of the values' position in the data string.

In the experiment, the height of the scanning plane is equal to about 1.3 meters from the ground, this leads to better results because the understory and other uninteresting objects below the scanning plane and the variation in the height of the scanning plane is assumed to be negligible in our experiment. The measurement range is limited to 8 m, which is not beyond the reachable workspace of the crane of the logging harvester and can echo sufficient laser echo data from a single trunk.

**Figure 4.**
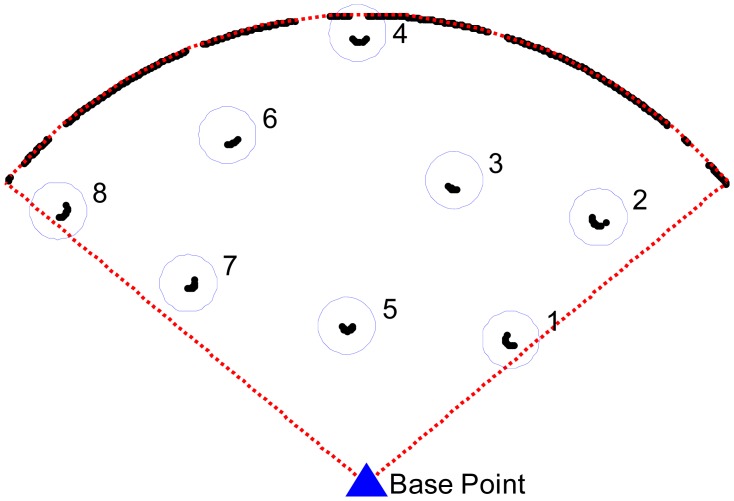
The scanning and clustering result of the experiment.

In polar form, supposing vector *D_i_* = [*l_i_, θ _i_*], where *i* = 1 to 401; *θ_i_* is the angular positions; *l_i_* is the horizontal distance value between the laser reflecting point and the measuring base point, then, *l_i_* can be calculated as following Equation (1):

(1) $$l_{i}=l_{i}^{\text{raw}}\times \cos\alpha \times \cos\beta$$

where $l_{i}^{\text{raw}}$ is the raw distance value of every scanning angular in polar form acquired directly from the laser scanner; *α* and *β* are the roll and pitch angle acquired by the AHRS. After one scan in the aspen forest, the laser scanning measurement data forms a point cloud in polar form as shown in Figure 4.

In Figure 4, the blue point is the measuring base point and the red dashed line is the maximum range (8 m) of the laser scanning plane. Some of the cloud points are clustered as shown in Figure 4, and each set of points inside a circle represents one cluster. In the scanning plane, the areas behind the nearest trunk are blind. During the logging operation, the harvester commonly works on the nearest trunk firstly, so the scanning blind areas can be neglected. A clustering and filtering algorithm can be used to filter out any uninteresting objects or incorrect data and extract the trunks from the point cloud.

### 3.2. Clustering, Filtering and Extracting the Trunk

The measurement data is processed in increasing order of the bearing angle from 40° to 140°. The cluster can be defined by two edge points, which are the measurement points that satisfy [6]:

(2) $$\left\|l_{i}-l_{i-1}\right\|=\Delta l>\Delta l_{\max}$$

where *l_i_* is the distance value of the *i*^th^ measurement; Δ*l*_max_ is the threshold for the allowed distance in distance inside a cluster. If the distance Δ*l* between two adjacent points is larger than Δ*l*_max_, one of points *i*^th^ and *I* − 1^th^ belongs to the cluster and the other one belongs to the background or other cluster. In our experiment, the distance between two trunks is large, so we choose Δ*l*_max_ = 0.2 m, then, eight clusters can be extracted from the point cloud in Figure 3.

Using Equation (2) for extracting trunks from the point cloud is certainly effective and sufficient in a forest with just a few bare tree trunks, but in a more complex environment, the laser beam may hit uninteresting things, branches or the ground, and because of divergence of the laser, incorrect measurement data may exist in the point cloud, therefore, filtering algorithms should be used to filter out the incorrect points or clusters from the point cloud, and accept only the trunk clusters. The filtering is performed for each cluster by testing it against following four criteria [6]:

1. The minimum and maximum curvatures of the whole cluster.
2. The minimum value for the curvature of a single point *cur_i_*.
3. The greatest acceptable width of the cluster.
4. The smallest acceptable depth of the cluster.

A point or cluster should be rejected if it fails any of the above tests. The minimum and maximum curvatures of the whole cluster in (1) prescribe the acceptable value of the trunk radius. The curvature of a single point *cur_i_* in (2) can be calculated as Equation (3) [12]:

(3) $${\text{cur}}_{i}=(l_{i+1}-l_{i})-(l_{i}-l_{i-1})=l_{i+1}-2l_{i-1}+l_{i}$$

**Figure 5.**
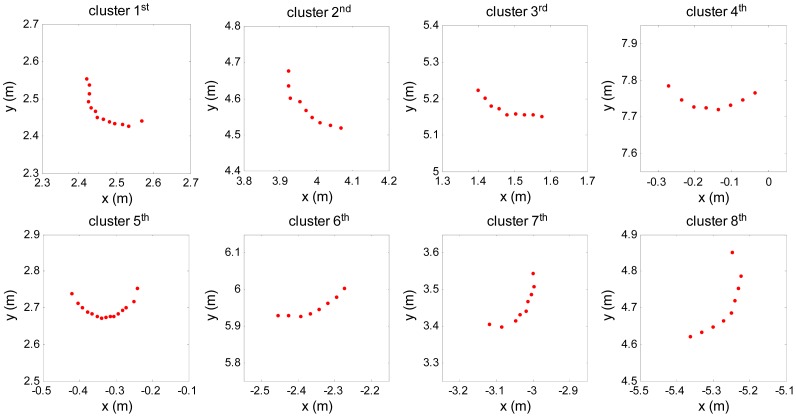
The point cloud in clusters after filtering.

The greatest acceptable width and the smallest acceptable depth of the cluster in (3) and (4) can filter out the ground or other uninteresting things. The vector ***D****_i_* = [*l_i_, θ_i_*] of the validated points can be transform to the vector $P_{i}=[p_{i}^{x},p_{i}^{y}]$ defined in rectangular form. Figure 5 gives point cloud of every cluster defined in rectangular form after filtering.

### 3.3. Calculating the Parameters of the Trunk

After filtering and extracting the trunk clusters from the point cloud, the trunk clusters offer a variety of features that can be used for the logging operation such as radius, center location and distances between adjacent trunks.

Supposing that the matrix the ***P*** = [*P*_1_, *P*_2_,…,*P_m_*] represents the position of every point in the truck cluster in rectangular form, *m* is the number of points in one cluster. On the assumption that all cross sections of the standing trees are approximately standard circles, there exist a number of different methods to fit a circle from the vector ***P****_i_* in a trunk cluster and to estimate the parameters of every trunk. In this paper, the effective conjugate gradient method is used.

Supposing the center location of the trunk is *O* = (*O_x_, O_y_*) and the radius of the trunk is *R*, then, the vector $P_{i}=[p_{i}^{x},p_{i}^{y}]$ satisfies:

(4) $$\begin{array}{c} {\left(p_{i}^{x}-O_{x}\right)}^{2}+{\left(p_{i}^{y}-O_{y}\right)}^{2}=R^{2}\\ \Rightarrow {\left(p_{i}^{x}\right)}^{2}+{\left(p_{i}^{y}\right)}^{2}-2p_{i}^{x}O_{x}-2p_{i}^{y}O_{y}+{\left(O_{x}\right)}^{2}+{\left(O_{y}\right)}^{2}=R^{2}\\ \Rightarrow 2p_{i}^{x}O_{x}+2p_{i}^{y}O_{y}+\left[R^{2}-{\left(O_{x}\right)}^{2}-{\left(O_{y}\right)}^{2}\right]={\left(p_{i}^{x}\right)}^{2}+{\left(p_{i}^{y}\right)}^{2} \end{array}$$

Supposing the unknown vector ***x*** = [*x*_1_, *x*_2_, *x*_3_]^T^ = [*O_x_, O_y_, R*^2^ – (*O_x_*)^2^ – (*O_y_*)^2^]^T^, the coefficients vector $\alpha_{i}={\left[\alpha_{i1},\alpha_{i2},\alpha_{i3}\right]}^{\text{T}}={\left[2p_{i}^{x},2p_{i}^{y},1\right]}^{\text{T}},$ and $b_{i}={\left(p_{i}^{x}\right)}^{2}+{\left(p_{i}^{y}\right)}^{2},$ then, the Equation (4) can be transformed as follows:

(5) $$a_{i1}x_{1}+a_{i2}x_{2}+a_{i3}x_{3}=b_{i}$$

Then the scanning points in one cluster form a set of linear algebraic equations with constant coefficients as in the following Equation (6):

(6) $$\{\begin{array}{c} a_{11}x_{1}+a_{12}x_{2}+a_{13}x_{3}=b_{1}\\ a_{21}x_{1}+a_{22}x_{2}+a_{23}x_{3}=b_{2}\\ \vdots \\ a_{m1}x_{1}+a_{m2}x_{2}+a_{m3}x_{3}=b_{m} \end{array}$$

In order to get the parameters of the trunk, it is desired to solve for the unknown quantities ***x*** = [*x*_1_, *x*_2_, *x*_3_]^T^, given the coefficients *α_ij_* for *i* = 1,2,…*m, j* = 1,2,3 and *b_i_* for *i* = 1,2,…,*m*. After solving the unknown quantities ***x***, the center location of the trunk *O* = (*O_x_*,*O_y_*) and the radius of the trunk *R* can be calculated as shown in Equation (7):

(7) $$\begin{array}{ccc} O_{x}=x_{1}; & O_{y}=x_{2}; & R=\sqrt{x_{1}^{2}+x_{2}^{2}+x_{3}} \end{array}$$

Equation (6) can be written in a vector-matrix form as Equation (8):

(8) Ax=b

where it is assumed that *A* ∈ ℛ^m×3^, *x* ∈ ℛ^3×1^, *b* ∈ ℛ^m×1^, and:

(9) $$\text{A}=\left[\begin{array}{ccc} a_{11} & a_{12} & a_{13}\\ \begin{array}{l} a_{21}\\ \cdots \end{array} & \begin{array}{l} a_{22}\\ \cdots \end{array} & \begin{array}{l} a_{23}\\ \cdots \end{array}\\ a_{m1} & a_{m2} & a_{m3} \end{array}\right]\begin{array}{cc} ;\text{x}=\left[\begin{array}{c} x_{1}\\ x_{2}\\ x_{3} \end{array}\right]; & \text{b}=\left[\begin{array}{c} b_{1}\\ b_{2}\\ \cdots \\ b_{m} \end{array}\right] \end{array}$$

For *m* > 3, Equation (8) is referred to as being over-determined (more equations than unknowns). In order to get parameters of the trunk for quick harvesting operations, the time constraints and accuracy for solving Equation (8) are important, so the Fletcher-Reeves conjugate gradient algorithm (F-R algorithm) is used to solve it. The F-R algorithm is a real time and online neuro-computing approach for solving system of linear algebraic equations and can achieve fast speed of convergence in neuro-computing. The F-R algorithm is given as follows [13,14]:

Supposing ***x****_k_* is the discrete-time iterative solution of Equation (8) using the F-R algorithm, *k* is the discrete-time index, then, the solution error for solving ***Ax*** = ***b*** is given by:

(10) e=Ax−b

The F-R algorithm can be incorporated into the discrete-time learning rule to derive an update expression for the iterate ***x****_k_*, the update of the solution is given by:

$${\text{x}}_{k+1}={\text{x}}_{k}+\alpha_{k}{\text{d}}_{k}$$

where

$$\alpha_{k}=\frac{-{\text{g}}_{k}^{\text{T}}{\text{d}}_{k}}{{\text{d}}_{k}^{\text{T}}{\text{A}}^{\text{T}}\text{A}{\text{d}}_{k}}$$

The vector ***d****_k_* is the current direction vector, and *ε*(**·**) is the object function to be determined, and is given by:

$$ɛ(\text{x})=\frac{1}{2}{\left\|\text{e}\right\|}_{2}^{2}=\frac{1}{2}{\text{e}}^{\text{T}}\text{e}$$

Therefore, the Fletcher-Reeves conjugate gradient algorithm (with restart) for solving ***Ax*** = ***b*** is summarized in the following steps [14]:

- Step 1: Set initial condition ***x***_0_.
- Step 2: Compute ***g****_k_*|*_k_*_=0_ = ***g***_0_ = ***A***^T^***Ax***_0_ – ***A***^T^***b***.
- Step 3: Set ***d***_0_ = −***g***_0_.
- Step 4: Compute ***x****_k_*_+1_ = ***x****_k_* + *α_k_****d****_k_*, where $\alpha_{k}=-g_{k}^{\text{T}}d_{k}/(d_{k}^{\text{T}}A^{\text{T}}Ad_{k})$
- Step 5: Compute ***g****_k_*_+1_ = ***A***^T^***Ax****_k_*_+1_ – ***A***^T^***b***.
- Step 6: Compute ***d****_k_*_+1_ = −***g****_k_*_+1_ + *β_k_****d****_k_*, where $\beta_{k}=g_{\text{k}+1}^{\text{T}}g_{\text{K}+1}/(g_{\text{k}}^{\text{T}}g_{\text{K}})$
- Step 7: If *k* < *k*_max_ go to step 4.
- Step 8: Continue until convergence is achieved; termination criterion could be ‖*d_k_*‖ < *ε* (where *ε* is an appropriate predetermined small number) and *k* > *k*_max_.

where *k*_max_ is the maximum number of iterations. In our experiment, *k*_max_ is set to 1,000 and the initial condition ***x*** = [0,0,0]^T^, consequently, faster convergence can be expected. The distance between two trunks in the horizontal plane can be calculated via the center locations of two trunks.

## 4. Results and Discussion

The trunk feature extracting process presented in the previous section is programmed with the Visual C++ 6.0 introduced by Microsoft and Matcom 4.5 introduced by MathWorks as MATLAB to a C++ compiler. All of the calculation results such as radii, location of the trunks and distances between adjacent trunks can be displayed on a human-computer interface for the operator.

**Figure 6.**
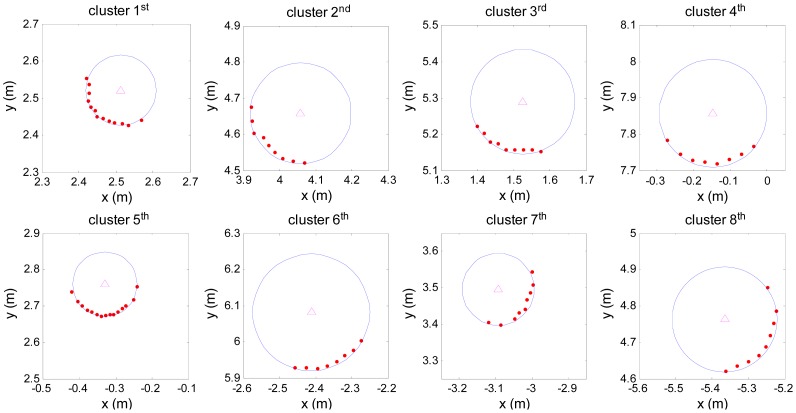
The calculation results.

The result of the calculations on the trunk parameter is given in Figure 6. There are eight blue circles which are the fitting results of the trunk point cloud, and the centre of the trunks are marked by the triangle. Supposing the measurement base point is the origin of the rectangular form, then the 2nd and 5th column of Table 1 give the manual measurement values of the central location and radius of the trunk, which are acquired manually by the range finder and vernier, respectively.

Extracting the parameters of the trunk from the point cloud also can be achieved by the Least Square Fitting algorithm (LSF algorithm) [15]. The 3rd and 6th column of Table 1 give the calculation results via the LSF algorithm. The 4th and 7th column of Table 1 give the calculation results via the F-R algorithm used in this paper.

Table 2 gives the maximum error and time consumption on the calculation by LSF and F-R algorithm respectively.

As shown in Table 2, the maximum error for the center location and radius are nearly the same by the LSF and F-R algorithm, but time consumption is reduced by the F-R algorithm. The errors are mainly caused by the resolution and systematic errors of the laser device and the approximation error of the F-R algorithm. The error increases as the distance between the base point of the laser scanner and the trunk increases. The max error and time consumption of the calculation meet the requirements for the accuracy (<15 cm) and real time (<10 ms) for logging harvesting operations.

## 5. Implementation

**Figure 7.**
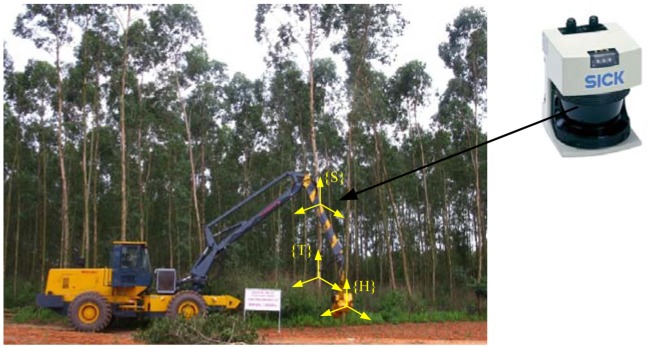
Implement on the logging harvester.

It is found by the experiments that the process of delimbing, peeling, and bucking can be completed fast by the logging harvester, but for the process of the alignment of the harvesting head to capture the trunk, the operator needs a lot of observation, judgment and repeated operations, which lead to time and fuel losses. In order to improve the operation efficiency and reduce the operating costs during the logging harvesting operations, the laser scanner and inertial measurement system presented in this paper can be mounted on the outer boom of the crane to determine the location of the trunk near the harvesting head as seen in Figure 7.

In Figure 7, the frames {S}, {H} and {T} are located at the base point of the laser scanner, the harvesting head, and the target trunk, respectively. The point cloud is collected for the surrounding trees and using the trunk feature extracting approach presented in Section 3, the pose and position matrix TST of the truck respective to the laser scanner base point can be obtained in every control period and transferred to the on-board computer system of the logging harvester.

Using the D-H parameters of the manipulator and combining the information of the harvesting head angle encoders, the pose and position matrix THS relating the harvesting head frame to the laser scanner base point can be determined [16].

Then, the pose and position matrix THTfor the trunk relative to the harvesting head can be expressed in the multiplication product of successive 4 × 4 homogeneous matrices as given by Equation (11):

(11) THT=TSTTHS

The trajectory of the crane for trunk capture is planned in Cartesian space. The harvesting head moves smoothly one step in every control period until reaching the target. The motion planning and control flow for trunk capturing process can be summarized by the following steps:

- Step 1: The laser scanner makes a fan-shaped scan of the surrounding area, and gets the location of every trunk in the scanning range.
- Step 2: The pose and position matrix TST of the truck respective to the measurement base point is formed. Every current joint angle *θ* of the crane and harvesting head is received from the angle encoder.
- Step 3: Using the Equation (11), the matrix THT for the target trunk relative to the harvesting head is formed.
- Step 4: Considering the constraints of joint velocity and acceleration, the central controller of the logging harvester plans the desired set points Δ*p_k_* of the Cartesian-coordinate trajectory for the harvesting head to catch the target trunk, and the number of the points *k* (10 < *k* < 100) is decided by the distance between the harvesting head and the target.
- Step5: Using the inverse kinematics solution, the angle increments Δ*θ* of every joint are acquired from the first planned set point Δ*p*_1_.
- Step 6: The angle increments Δ*θ* are sent to the hydraulic controllers respectively, and the hydraulic controllers guide the motion of every hydraulic cylinder.
- Step 7: In the next ten control periods, the angle increments are acquired from the planned set points similarly, and hydraulic cylinders move according to the angle increments.
- Step 8: if the capture range of the target trunk is reached, the gripper is closed, and finishes the mission, otherwise, it returns to step 1.

Because of the vibration of crane and control errors, only the first ten planned set points can be used for the task from step 4 to step 7, the rest of the planned set points should be discarded, and a new path planning calculation should be made in the next control period.

## 6. Conclusions and Outlook

In this paper, in order to realize semi-automated logging harvesting, the point cloud for standing trees is collected with a 2D laser scanner. A cluster extracting algorithm and filtering algorithm is used to classify each trunk from the point cloud. The radii and positions of the trees are calculated by the Fletcher-Reeves conjugate gradient algorithm. Compared with previous work by other researchers, the calculation time consumption is reduced. The implementation of the calculation result on a logging harvester is discussed in particular.

The aim of this research relates to the human aspect of logging harvester operations. Logging harvesters are difficult to control and operator training is time consuming and expensive. If the location of every trunk relative to the harvesting head is known by the laser scanner, the operator could just indicate which tree to cut and the crane would automatically grasp it. The burden of the operator could be lightened by increasing the automation level of the logging harvester in the future.

## Figures and Tables

**Table 1.** The parameters of the trunks acquired by different methods.

**No.**	**Center location (*O****_x_*,***O****_y_***) (cm)**			**Radius (cm)**		

	**Manual**	**LSF algorithm**	**F-R algorithm**	**Manual**	**LSF algorithm**	**F-R algorithm**
1	(247.0, 248.0)	(251.4, 252.1)	(251.4, 252.1)	8.6	9.9	9.9
2	(397.9, 455.7)	(405.9, 465.8)	(405.9, 465.8)	13.5	14.7	14.7
3	(149.1, 520.0)	(152.6, 529.0)	(152.6, 529.0)	13.6	15.2	15.2
4	(−15.2, 782.9)	(−14.7, 785.9)	(−14.7, 785.8)	13.3	15.6	15.5
5	(−32.8, 272.0)	(−33.0, 276.0)	(−33.0, 276.0)	9.0	9.3	9.3
6	(−237.0, 597.7)	(−241.3, 608.5)	(−241.2, 608.2)	14.6	17.3	17.0
7	(−305.4, 346.7)	(−309.3, 349.6)	(−309.3, 349.6)	10.3	10.4	10.4
8	(−532.5, 471.1)	(−536.5, 476.3)	(−536.5, 476.3)	13.9	15.2	15.2

**Table 2.** The performance comparison of LSF algorithm and F-R algorithm.

**Characteristic**	**LSF algorithm**	**F-R algorithm**
Max error on the center location (cm)	(8.0, 10.8)	(8.0, 10.5)
Max error on the radius (cm)	2.7	2.4
Time consumption (ms)	0.28	0.19
